# Analyzing the nexus between burnout and psychological distress in pediatric oncology nurses: a descriptive correlational investigation

**DOI:** 10.1186/s12912-025-03061-5

**Published:** 2025-04-28

**Authors:** Wafaa Osman Abd El-Fatah, Taghred Mohamed, Nahla Abdallah, Heba Mahmoud Ahmed, Sorayia Ramadan Abd El Fattah Ayaad

**Affiliations:** 1 Faculty of Nursing, Helwan University, Cairo, Egypt; 2 Faculty of Medicine, Al-Azhar University, Cairo, Egypt; 3 Cairo, Egypt; 4 Faculty of Nursing, 6 October University, and Ain Shams University, Cairo, Egypt

**Keywords:** Burnout, Psychological distress, Pediatric oncology nurses, Occupational stress, Nurse well-being, Work-life balance

## Abstract

**Background:**

Burnout and psychological distress are significant concerns among healthcare professionals, particularly pediatric oncology nurses, who face emotional and physical challenges due to their demanding roles. Continuous exposure to life-threatening illnesses, high patient mortality rates, and emotionally taxing interactions contribute to severe occupational stress, potentially affecting both nurse well-being and patient care quality. This study aimed to analyze the relationship between burnout and psychological distress among pediatric oncology nurses at the Children’s Cancer Hospital Foundation (CCHE 57357) in Egypt.

**Methods:**

A descriptive correlational research design was employed, involving a purposive sample of 188 nurses working in inpatient and intensive care units. Data were collected using three validated tools: (1) a Sociodemographic Data Sheet, (2) the Copenhagen Burnout Inventory (CBI) to assess personal, work-related, and client-related burnout, and (3) the Kessler Psychological Distress Scale (K10) to measure levels of psychological distress. The instruments underwent translation and validation for use in Arabic. Data collection occurred over six months, with statistical analysis conducted using SPSS (version 25). Descriptive statistics, Pearson correlation, and linear regression models examined relationships between burnout and psychological distress.

**Results:**

The findings revealed that 55.8% of nurses had low burnout levels, 31.4% had moderate burnout, and 12.8% exhibited high burnout. In addition, psychological distress levels varied, with 34% experiencing moderate distress, 25% showing very high psychological distress, and 22% reporting high distress. A statistically significant positive correlation (*r* = 0.59, *p* = 0.00) was identified between total burnout and psychological distress, indicating that higher burnout levels were associated with increased psychological distress. Regression analysis further confirmed the significant impact of psychological distress on burnout (R² = 0.35, Beta = 0.59, *p* = 0.00).

**Conclusion:**

This study highlights the significant correlation between burnout and psychological distress among pediatric oncology nurses, emphasizing the critical need for specialized interventions to promote mental well-being. The findings stress the importance of implementing stress reduction programs, fostering better work-life balance strategies, and providing comprehensive mental health support to alleviate burnout and distress. Effectively addressing these issues will not only improve nurses’ overall well-being but also contribute to enhancing the quality of care in pediatric oncology settings.

**Clinical trial registration:**

Not applicable.

## Introduction

Pediatric oncology nursing is one of the most emotionally and physically demanding specialties within the healthcare profession. Nurses in this field provide critical care to children diagnosed with cancer, often witnessing intense suffering and, in some cases, the loss of young lives. The nature of pediatric oncology care requires a high level of technical proficiency, emotional resilience, and the ability to provide psychological support to patients and their families. This constant exposure to distressing situations and the emotional labor involved significantly contribute to occupational stress, burnout, and psychological distress among nurses [1].

Burnout is a psychological syndrome that results from chronic workplace stress that has not been successfully managed. It is characterized by three primary dimensions: emotional exhaustion, depersonalization, and a diminished sense of personal accomplishment [2]. Emotional exhaustion manifests as extreme fatigue and an inability to engage effectively with work. Depersonalization refers to a sense of detachment from patients, leading to cynical or indifferent attitudes. A reduced sense of personal accomplishment is marked by feelings of inefficacy and dissatisfaction with one’s professional contributions [3]. These factors not only affect individual nurses but also have profound implications for patient care, hospital functioning, and overall healthcare service quality [4, 5].

Psychological distress, a broad term encompassing anxiety, depression, and other emotional disturbances, frequently coexists with burnout [6]. It is particularly prevalent among pediatric oncology nurses, who routinely engage in high-stress environments that demand both technical expertise and significant emotional engagement. Research suggests that prolonged psychological distress can lead to serious mental health conditions, decreased job performance, absenteeism, and high turnover rates [7]. Moreover, nurses experiencing high levels of burnout and psychological distress may inadvertently compromise patient safety, leading to lower quality of care and adverse patient outcomes [8].

The demanding nature of pediatric oncology nursing is further exacerbated by factors such as excessive workload, long working hours, staff shortages, and inadequate institutional support [9]. A study conducted among oncology nurses indicated that those working extended shifts and handling a high volume of critically ill patients reported significantly higher levels of burnout compared to their counterparts in less intensive settings [10]. Similarly, research has demonstrated that inadequate coping mechanisms and insufficient organizational support can aggravate the psychological burden experienced by nurses [11]. These stressors, if not properly managed, can contribute to emotional exhaustion, further affecting nurses’ ability to provide compassionate and effective care [12].

The relationship between burnout and psychological distress has been well-documented, with numerous studies establishing a significant correlation between the two [13]. In pediatric oncology nursing, this connection is particularly critical, given the high-stakes nature of their work. Nurses who experience burnout often exhibit symptoms of anxiety, depression, and emotional fatigue, which, in turn, affect their professional efficacy and patient interactions [14]. This cyclical relationship can lead to a downward spiral, wherein psychological distress exacerbates burnout, and burnout intensifies psychological distress, resulting in an increasingly compromised workforce [15].

A major contributor to burnout and psychological distress among pediatric oncology nurses is their frequent exposure to patient suffering and death [16]. Providing end-of-life care to children is a deeply distressing experience that demands both technical expertise and emotional resilience. Many nurses struggle with the emotional toll of witnessing young patients endure pain, undergo aggressive treatments, and, in some cases, succumb to their illnesses. This continuous exposure to grief and loss without adequate emotional support mechanisms can significantly elevate levels of burnout and psychological distress [17].

While burnout and psychological distress are prevalent across various nursing specialties, pediatric oncology nurses face unique challenges that intensify these conditions. Studies indicate that nurses working in oncology settings experience higher rates of emotional exhaustion and compassion fatigue compared to those in general medical or surgical units [18]. This is often attributed to the chronic exposure to pediatric cancer patients, complex treatment regimens, and the emotional labor required to support patients and families during highly distressing moments [19]. The emotional burden of these responsibilities is further compounded by systemic issues such as staff shortages, limited mental health resources, and inadequate institutional recognition of the psychological impact of their work [20].

Addressing burnout and psychological distress among pediatric oncology nurses requires a multifaceted approach. Hospitals and healthcare institutions must prioritize nurse well-being by implementing structured mental health programs, providing access to psychological counseling, and fostering a supportive work environment [21]. Regular mental health screenings, stress management workshops, and peer support groups have been shown to significantly alleviate stress and reduce burnout rates among healthcare professionals [22]. Additionally, promoting work-life balance through flexible scheduling, adequate staffing, and professional development opportunities can help mitigate the effects of occupational stress [23].

Another critical aspect of intervention is enhancing nurses’ resilience and coping mechanisms. Training programs that focus on emotional resilience, mindfulness-based stress reduction, and cognitive-behavioral strategies have demonstrated efficacy in reducing burnout and improving overall psychological well-being [24]. Encouraging self-care practices, such as mindfulness exercises, regular physical activity, and professional debriefing sessions, can further support nurses in managing the emotional challenges associated with their work [25]. Additionally, fostering a workplace culture that acknowledges and validates the emotional experiences of nurses can contribute to a healthier and more sustainable work environment [26].

The significance of studying burnout and psychological distress in pediatric oncology nurses extends beyond individual well-being. The quality of patient care is directly influenced by the mental and emotional state of nurses. A workforce experiencing high levels of burnout and psychological distress is more likely to make medical errors, experience diminished empathy, and exhibit decreased job performance. Moreover, an Egyptian study explained that 43.2% of nurses had a moderate level of burnout, and 32.6% had a high level of burnout; 54.6% of nurses had average levels of emotional exhaustion, 48% scored high on depersonalization, and 77.5% showed significant reductions in personal accomplishment [18, 27, 47]. Thus, recognizing and addressing these issues is essential not only for improving nurse well-being but also for ensuring optimal patient care and safety.

## Methods

### The aim of the study

The aim was to examine the relationship between burnout and psychological distress among pediatric oncology nurses.

**Research questions**:

1. What are burnout levels among pediatric oncology nurses?
2. What are the psychological distress levels among pediatric oncology nurses?
3. Is there a relationship between burnout and psychological distress among pediatric oncology nurses?

### Study design

This study utilized a descriptive correlational research design to examine the relationship between burnout and psychological distress among pediatric oncology nurses. A descriptive correlational approach was chosen because it allows for the exploration of relationships between variables without manipulation or intervention. This design is well-suited for healthcare research, where ethical concerns often prevent the experimental manipulation of work-related stressors. It enables the identification of associations between burnout and psychological distress in a naturalistic healthcare setting, providing valuable insights into their prevalence and impact.

### Study setting

The study was conducted at the Children’s Cancer Hospital Foundation (CCHE 57357) in Egypt, the largest pediatric oncology hospital in the region. CCHE 57,357 is a specialized cancer center with six floors and a total of 320 beds distributed across various departments, including five inpatient units, outpatient clinics, an emergency department, two intensive care units (ICUs), a bone marrow transplantation unit, diagnostic radiology services, and a research department. This setting was selected because it provides comprehensive pediatric oncology care, ensuring a high concentration of nurses who work in emotionally and physically demanding environments. Given the nature of the hospital’s operations, its staff frequently encounters distressing situations such as pediatric patient mortality and long-term treatment processes, making it an ideal location for studying burnout and psychological distress.

### Sample and sampling

A purposive sampling technique was used to recruit nurses working in pediatric oncology care at CCHE 57,357. Using a purposive sampling method is a more effective strategy for enhancing data reliability and ensuring the validity of the study. This approach enables researchers to select participants with firsthand experience in burnout and psychological distress within pediatric oncology environments. Given that pediatric oncology nurses constitute a specialized and relatively small subgroup within the nursing profession, their accessibility is often limited. The demanding nature of their work schedules further complicates the recruitment of a random sample. By directly identifying and engaging eligible participants, purposive sampling streamlines the data collection process, making it more efficient. The study included 188 nurses who met the inclusion criteria, ensuring they had direct experience in inpatient care or intensive care units (ICUs).

The inclusion criteria were:

- Professional Experience: Nurses with at least one year’s experience in pediatric oncology.
- Employment in Critical Units: Nurses working in inpatient units and ICUs were prioritized, as these settings involve continuous patient care and high emotional demands.

The exclusion criteria were:

- Nurses who suffer from chronic diseases such as diabetes and high blood pressure or any psychological disorders such as anxiety, etc., were excluded by asking them to self-report.

The sample size was determined using a statistical formula for correlational research, ensuring adequate power for detecting meaningful relationships between burnout and psychological distress. A total of 188 nurses participated, providing a sufficiently large and diverse sample for statistical analysis. The sampling strategy ensured that the findings would be representative of the broader pediatric oncology nursing population while minimizing biases related to nurse selection.

### Data collection tools

Three main tools were utilized to gather comprehensive data on nurses’ sociodemographic backgrounds, burnout levels, and psychological distress. These instruments were selected based on their robust psychometric properties, widespread usage in healthcare research, and relevance to the constructions under investigation.

1. This sheet was developed by the researchers to collect personal information, including age, gender, educational level, years of experience, marital status, place of residence (urban or rural), monthly income sufficiency, and number of children. Gathering this background data helped contextualize the findings and allowed for the examination of potential relationships between demographic factors, burnout, and psychological distress.
2. The Copenhagen Burnout Inventory (CBI) was developed by Kristensen and colleagues in 2005 [28]. It aims to measure burnout in three distinct dimensions: personal burnout, work-related burnout, and client-related burnout. The personal burnout subscale assesses the extent to which individuals experience physical and psychological exhaustion, while the work-related burnout subscale focuses on the exhaustion nurses attribute directly to their job responsibilities. The client-related burnout subscale measures exhaustion specifically linked to patient care. Each dimension comprises items scored using a Likert-style format, generally converted into values ranging from 0 (no burnout) to 100 (severe burnout). Higher scores indicate heightened burnout levels, although the CBI can also be treated as a continuous measure to detect subtle variations in burnout severity. Regarding validity and reliability, the CBI has shown strong internal consistency, with Cronbach’s alpha frequently exceeding 0.8 in multiple studies. In its original form, the instrument underwent a thorough psychometric evaluation, indicating excellent construct and convergent validity. The CBI has been translated into several languages, including Arabic, following rigorous forward-backward translation protocols to ensure conceptual equivalence. Before use, the Arabic version was pilot-tested to confirm clarity and cultural appropriateness, enhancing face validity in this specific context.
3. The Kessler Psychological Distress Scale (K10), introduced by Kessler and colleagues in 2003, was employed to assess the level of nonspecific psychological distress among participants [29]. It includes ten items, each rated on a five-point scale that ranges from “none of the time” (score 1) to “all of the time” (score 5). This tool captures the frequency of negative emotional states (e.g., nervousness, hopelessness) over the preceding four weeks. The overall score ranges from 10 to 50, with higher scores denoting higher levels of psychological distress. Numerous validation studies have demonstrated its high internal consistency, with Cronbach’s alpha values commonly around 0.9 or above, reflecting its reliability. Construct validity is evidenced by significant correlations with depression and anxiety measures. Like the CBI, the K10 has been translated into Arabic using forward-backward translation procedures; subsequent pilot testing verified that the final Arabic version retained both conceptual and linguistic fidelity. In the present investigation, the Arabic K10 was found to be comprehensible and relevant, supporting its content validity in an Egyptian cultural setting.

**Cronbach’s alpha values** for **the Copenhagen Burnout Inventory (CBI)**

**Table:** 

CBI subscale	Cronbach’s Alpha (α)
Personal burnout	0.902
Work-related burnout	0.774
Client-related burnout	0.817

### Data collection procedure

Data was collected over six months, from April to October 2023, during the morning shifts. Two days each week—Sundays and Thursdays—were dedicated to this process. The researchers introduced themselves to potential participants, explained the purpose of the study in clear terms, and provided them with an overview of their rights, including the ability to withdraw at any time without consequence. Each participant was assigned a unique coded identifier instead of using names or other identifiable details, and access to the data was restricted to authorized research personnel only. Once written informed consent was obtained, participants were given the study instruments—comprised of the sociodemographic sheet, the CBI, and the K10—and were asked to complete them in a quiet area, typically within 20 to 30 min. The researchers were available to answer clarifying questions while also ensuring minimal disruptions during the data collection sessions.

### Data analysis

Upon completion of data collection, all responses were coded and entered into the Statistical Package for the Social Sciences (SPSS) for Windows (version 25). Both descriptive and inferential statistics were utilized. Descriptive analyses included frequency distributions, means, and standard deviations to characterize the sample and capture overall trends. Inferential statistical tests, such as Pearson’s correlation and linear regression, examined potential relationships and predictive effects between total burnout and total psychological distress. A significance level of *p* < 0.05 was used to determine statistical relevance. Data cleaning procedures ensured accuracy, while the normality of the data distribution was evaluated before advanced analyses.

### Ethical considerations

Ethical clearance was initially secured from the Children’s Cancer Hospital Foundation (CCHE 57357) as well as from the Scientific Research Ethical Committee of the Faculty of Nursing, Helwan University. Each participant received a clear explanation of the study aims, procedures, potential benefits, and any perceived risks. Participants then signed an informed consent form, acknowledging their voluntary participation. Confidentiality was maintained by assigning numeric codes to complete questionnaires, and all data were stored in locked files accessible only to the research team. The study adhered to the principles of the Declaration of Helsinki, ensuring respect for autonomy, privacy, and welfare of all participants. No financial or other inducements were offered, and participants were assured that study findings would be used strictly for research and improvement of nursing practices.

## Results

**Fig. 1:**
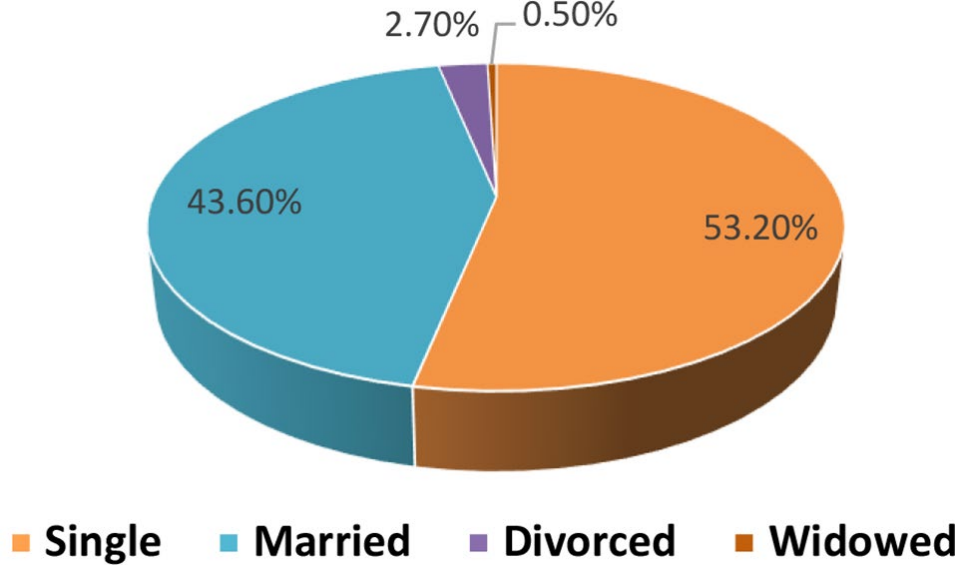
Distribution of nurses’ social status *n* = (188)

**Fig. 2:**
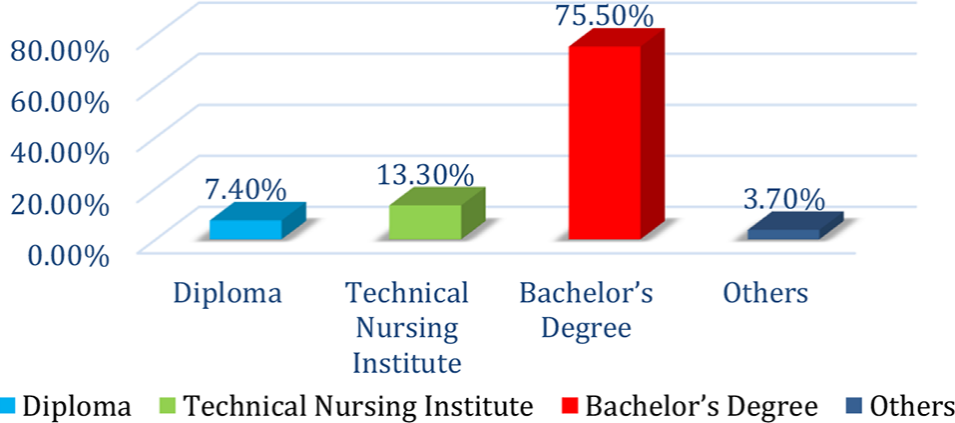
Distribution of nurses’ education levels *n* = (188)

**Fig. 3:**
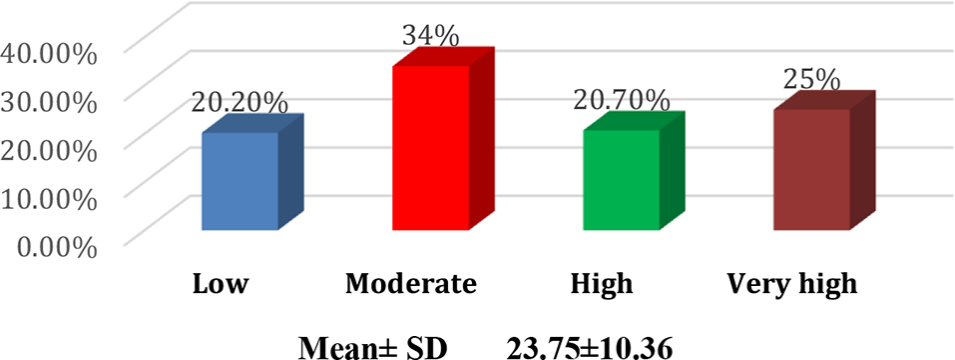
Distribution of nurses’ total psychological distress levels *n* = (188)

**Fig. 4:**
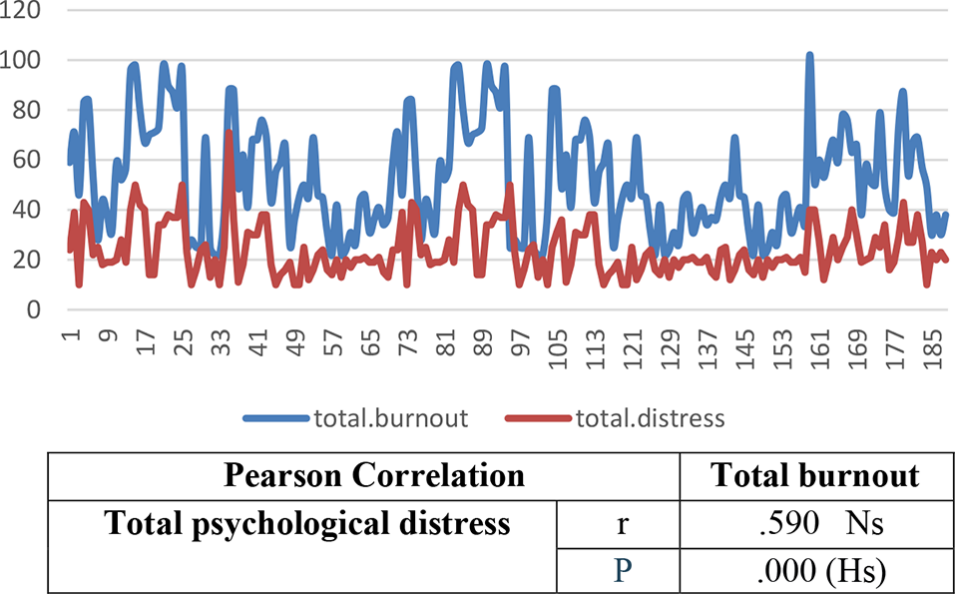
Correlation between nurses total burnout and total psychological distress *n* = (188)

- Table 1: Reveals that 63.8% of the nurses were female, 70.7% were aged between 20 and 30, and 61.7% had 1–5 years of experience at the current hospital. Additionally, 78.7% of the nurses worked 8–12 h/ day, 87.2% were employed in inpatient departments, and 53.2% had attended a stress management lecture. Furthermore, 61.7% of the nurses resided in urban areas, 52.7% reported having a sufficient monthly income, 39.4% had children, and 14.4% had two or more children.
- Figure 1: Shows that more than half of the staff nurses are single, more than two-fifths are married, 2.7% are divorced, and only 0.5% are widowed.
- Figure 2: Indicates that three-quarters of the staff nurses hold bachelor’s degrees, only 13% have completed technical institutes, 7% possess diplomas, and 4% have other qualifications.
- Table 2: Concludes that more than half of nurses have a low burnout level, more than one-third have a moderate burnout level, and 12.8% have a high burnout level.
- Figure 3: The data clarifies that 34% of the nurses experienced moderate psychological distress levels, one-quarter had extremely high psychological distress, and slightly more than one-fifth had high psychological distress. Additionally, one-fifth of the nurses reported low psychological distress levels.
- Figure 4: Illustrates a positive correlation between total burnout and total psychological distress as both increase and decrease always. The findings conclude that there was a highly significant positive correlation (*r* = 0.59, *p* = 0.00) between total burnout and total psychological distress among nurses.
- Table 3: Indicates a significant positive impact (R² = 0.35, Beta = 0.59, *p* = 0.00) of psychological distress on burnout among staff nurses.

**Table 1:** Distribution of nurses studied according to their socio-demographic characteristics (*n* = 188)

Items	No.	%
**1-Gender:**		
Male	68	36.2
Female	120	**63.8**
**2-Age:**		
20- ≤ 30	133	**70.7**
30 – ≤ 40	16	8.5
40 – ≤ 50	36	19.1
> 50	3	1.6
Mean ± SD 27.30 ± 3.02		
**3-Years of work experience in your hospital:**		
1–5 years	116	**61.7**
From 6–10 years	36	19.1
˃10 years	36	19.1
**4-Daily working hours:**		
> 8 h	40	21.3
From 8 to 12 h	148	**78.7**
**5-Your department:**		
Inpatient	164	87.2
ER	16	8.5
ICUs	8	4.3
**6-The previous courses and lecture:**		
Stress management	100	53.2
Motivation	44	23.4
Emotional intelligence	5	2.7
**7-Place of residence:**		
Urban	116	61.7
Rural area	72	38.3
**8-Your monthly income is:**		
Sufficient	99	52.7
Insufficient	89	47.3
**9-Have any children:**		
Yes	74	39.4
No	114	60.6
**10-Number of children:**		
One child	20	10.6
Two children	27	14.4
Three or more children	27	14.4

**Table 2:** Distribution of total staff nurses’ burnout levels (*n* = 188)

Total burnout	No.	%
Low	105	55.8
Moderate	59	31.4
High	24	12.8
Mean ± SD	52.16 ± 21.38	

**Table 3:** Linear regulation model of total burnout on total psychological distress among nurses

Linear regression model	*R* square	Unstandardized coefficients		Standardized coefficients	t	Sig.
		B	Std. Error	Beta		
(Constant)	**0.35**	23.262	3.165		7.351	0.000
Total psychological distress		1.217	0.122	**0.590**	9.961	0.000

a. Dependent variable: total burnout

## Discussion

The findings of this study provide important insights into the prevalence of burnout and psychological distress among pediatric oncology nurses at CCHE 57,357 in Egypt. The results highlight a significant positive correlation between burnout and psychological distress, emphasizing the urgent need for targeted interventions to mitigate these issues in the healthcare workforce.

### Burnout and psychological distress in pediatric oncology nurses

The study found that more than half of the nurses exhibited low levels of burnout, while a significant portion (31.4%) experienced moderate burnout levels, and 12.8% had high burnout levels. Similarly, psychological distress levels were moderate in 34% of the sample, while 25% exhibited extreme psychological distress. These findings align with previous research indicating that pediatric oncology nurses face significant emotional and physical stressors, leading to burnout and distress over time [30, 31]. The nature of pediatric oncology care, which involves long-term treatment regimens, exposure to patient suffering, and end-of-life care, contributes significantly to these stressors [32].

The statistically significant correlation (*r* = 0.59, *p* = 0.00) between burnout and psychological distress suggests that as burnout levels increase, psychological distress also rises. This is consistent with previous studies indicating a bidirectional relationship between these two factors, where prolonged exposure to workplace stress leads to emotional exhaustion, depersonalization, and reduced personal accomplishment, all of which exacerbate psychological distress [33, 34]. These findings reinforce the Job Demands-Resources (JD-R) model, which posits that excessive job demands without adequate resources contribute to burnout and emotional strain [35].

### Factors contributing to burnout and psychological distress

Several factors appear to contribute to the burnout and distress experienced by nurses in this study. The results indicate that nurses working in inpatient departments and ICUs had higher levels of burnout and psychological distress than those working in other departments. This aligns with international research, which has shown that critical care and oncology nurses are at heightened risk due to continuous exposure to life-threatening conditions, high patient mortality rates, and heavy workloads [36, 37].

The study also found that 78.7% of the nurses worked between 8 and 12 h per day, and long working hours have been repeatedly linked to burnout in healthcare settings [38]. Heavy workloads, emotional strain, and inadequate rest periods increase the likelihood of chronic exhaustion, negatively affecting both mental health and job performance [39].

Interestingly, more than half of the nurses had attended stress management lectures, yet a substantial proportion still reported burnout and distress. This suggests that while training programs may offer some relief, they may not be sufficient without systemic changes in workplace policies, workload distribution, and mental health support [25]. Previous studies suggest that interventions such as structured peer support programs, regular psychological debriefing, and flexible scheduling are more effective in reducing burnout in high-stress environments [40].

### Impact on nursing performance and patient care

Burnout and psychological distress have well-documented consequences on both nurse’s well-being and patient care quality. The results of this study support previous findings that indicate burnout among nurses leads to reduced job satisfaction, increased absenteeism, and higher turnover rates [41]. Additionally, nurses experiencing high psychological distress often report difficulties in maintaining focus, engaging empathetically with patients, and managing professional responsibilities [42]. The emotional toll of pediatric oncology nursing, particularly in end-of-life care situations, can further compound these issues, leading to compassion fatigue and emotional detachment from patients [43].

Moreover, psychological distress is linked to lower levels of patient safety and care quality, as nurses experiencing distress may be more prone to errors, reduced attentiveness, and impaired decision-making [44]. Ensuring that nurses receive adequate psychological support and manageable workloads is therefore crucial not only for their well-being but also for improving patient outcomes and maintaining high standards of care.

The findings of this study are consistent with global research on burnout and psychological distress in oncology nursing. A study conducted in the United States reported that more than 40% of oncology nurses experience moderate to severe burnout, with significant impacts on job retention and mental health [45]. Similarly, research from Saudi Arabia and European countries has demonstrated that oncology nurses are among the highest-risk groups for psychological distress due to their prolonged exposure to terminal illness and suffering [46].

However, the reported burnout levels in this study (12.8% high burnout, 31.4% moderate burnout) appear somewhat lower than those in other studies, where severe burnout rates have been documented as high as 30–40% (20). This difference may be attributed to hospital policies at CCHE 57,357, which provide stress management training and psychological support initiatives. Additionally, cultural factors, workplace norms, and social support systems may influence how burnout manifests among Egyptian nurses compared to their counterparts in Western healthcare systems. The researchers’ point of view is high workload: Due to the critical nature of pediatric oncology care, nurses must cope with long working hours, intense emotional stress, and high expectations from patients and their families, which increases stress and burnout. Also, dealing with critical care and death: continuously dealing with children with cancer and potential deaths can lead to severe emotional stress, increasing levels of burnout and emotional exhaustion. So, if job demands are high without adequate resources, this will lead to increased levels of burnout and psychological stress among nurses. Conversely, if job demands are balanced with supportive resources such as training, psychological support, and professional motivation, this may lead to improved job satisfaction and reduced burnout, thus enhancing the quality of care provided to patients. by conclusion The JD-R model helps explain the relationship between workload and burnout in pediatric oncology nursing. By analyzing how job stress and available resources influence each other, effective strategies can be developed to improve the work environment and reduce burnout, thereby enhancing nurses’ quality of life and healthcare efficiency.

### Limitations and advantages

There are several limitations associated with this study that should be acknowledged. First, its findings are specific to the group of nurses included in the sample, which may limit their applicability to other healthcare settings, populations, or regions. Second, reliance on self-reported data introduces the potential for bias, as participants might overstate or understate their experiences due to personal perceptions, memory recall, or social desirability. anonymous questionnaires: used to encourage participants to provide more honest answers without fear of outside judgment. Third, the study does not explore additional factors, such as workplace policies, individual coping mechanisms, or external support systems that could influence levels of burnout and psychological distress. We suggest triangulation with observational data as a mitigation strategy and proposed qualitative methods to explore workplace policies further.

Despite these limitations, the study offers valuable contributions. It provides a clearer understanding of the prevalence of burnout and psychological distress among nurses and the statistically significant relationship between the two. These findings contribute to the growing evidence of healthcare workforce challenges and stressors. The study also has practical implications, emphasizing the necessity of developing interventions to support nurses’ mental health and well-being. Furthermore, it raises awareness about the psychological demands by nurses, highlighting the importance of ongoing research and targeted resource allocation to address these issues effectively.

## Conclusion

Based on the results of the current study,

The study found that over 50% of nurses reported low levels of burnout, while more than 30% experienced moderate levels of psychological distress. A statistically significant relationship was observed between total burnout and psychological distress, suggesting the importance of addressing these issues. Implementing targeted interventions could support nurses’ mental health and overall well-being, which may positively impact patient care and workforce sustainability.

### Recommendations

Based **on the findings of the current study, the following recommendations are proposed**:

1. Routine mental health assessments and evaluations to detect early signs of burnout and psychological distress.
2. Further research involving a larger sample of oncology nurses from diverse healthcare settings.
3. Additional enhancement of nurses’ quality of life to improve the care provided to child patients.
4. Enhancing Institutional Support: Train leaders to recognize and address burnout and implement recognition and incentive programs for staff.

## Data Availability

the same data availability applicablethe datasets used and/or analyzed during the current study are available from the corresponding author upon reasonable request.
